# Effect of Erythropoietin, Iron Deficiency and Iron Overload on Liver Matriptase-2 (TMPRSS6) Protein Content in Mice and Rats

**DOI:** 10.1371/journal.pone.0148540

**Published:** 2016-02-04

**Authors:** Jana Frýdlová, Petr Přikryl, Jaroslav Truksa, Lucas L. Falke, Xin Du, Iuliia Gurieva, Martin Vokurka, Jan Krijt

**Affiliations:** 1 Institute of Pathophysiology, Charles University in Prague, First Faculty of Medicine, Prague, Czech Republic; 2 Institute of Biotechnology, Laboratory of Tumour Resistance, Academy of Sciences of the Czech Republic, Prague, Czech Republic; 3 Department of Pathology, Kidney Group, University Medical Center Utrecht, Utrecht, the Netherlands; 4 Division of Medical Genetics, Department of Medicine, University of California San Diego, La Jolla, California, United States of America; Lady Davis Institute for Medical Research/McGill University, CANADA

## Abstract

Matriptase-2 (TMPRSS6) is an important negative regulator of hepcidin expression; however, the effects of iron overload or accelerated erythropoiesis on liver TMPRSS6 protein content *in vivo* are largely unknown. We determined TMPRSS6 protein content in plasma membrane-enriched fractions of liver homogenates by immunoblotting, using a commercial antibody raised against the catalytic domain of TMPRSS6. Plasma membrane-enriched fractions were obtained by centrifugation at 3000 g and washing. TMPRSS6 was detected in the 3000 g fraction as a 120 kDa full-length protein in both mice and rats. Feeding of iron-deficient diet as well as erythropoietin treatment increased TMPRSS6 protein content in rats and mice by a posttranscriptional mechanism; the increase in TMPRSS6 protein by erythropoietin was also observed in *Bmp6*-mutant mice. Administration of high doses of iron to mice (200, 350 and 700 mg/kg) decreased TMPRSS6 protein content. Hemojuvelin was detected in the plasma membrane-enriched fractions of control animals as a full length protein of approximately 52 kDa; in iron deficient animals, the full length protein was partially cleaved at the N-terminus, resulting in an additional weak band of approximately 47 kDa. In livers from hemojuvelin-mutant mice, TMPRSS6 protein content was strongly decreased, suggesting that intact hemojuvelin is necessary for stable TMPRSS6 expression in the membrane. Overall, the results demonstrate posttranscriptional regulation of liver TMPRSS6 protein by iron status and erythropoietin administration, and provide support for the interaction of TMPRSS6 and hemojuvelin proteins *in vivo*.

## Introduction

Iron deficiency is the most common cause of anemia worldwide. Although most cases of iron deficiency anemia respond to oral iron supplementation, some patients suffer from iron-refractory iron deficiency anemia, which is refractory to dietary iron, and can be only partly corrected by parenteral iron administration [1]. Seven years ago it was discovered that this anemia can be caused by mutations in the *TMPRSS6* gene [2–6] which result in inappropriately high expression of hepcidin, the key iron-regulatory hormone [7]. Elevated hepcidin blocks iron export from enterocytes and macrophages, thus limiting iron availability for erythropoiesis.

The newly discovered role of *TMPRSS6* in iron metabolism has raised questions both about its mode of action, as well as about its physiological regulation. *TMPRSS6* encodes a serine protease, matriptase-2 (TMPRSS6), which has relatively low substrate specificity [8]. It has been convincingly demonstrated that, *in vitro*, TMPRSS6 cleaves hemojuvelin (HFE2), a GPI-bound hepatocyte membrane component of the hepcidin regulatory pathway [9]. HFE2 is a coreceptor for bone morphogenetic protein 6 (BMP6), which is transcriptionally induced by iron overload [10], and, through interaction with BMP receptors, activates phosphorylation of SMAD proteins and ultimately increases transcription of hepcidin [11]. Therefore, it is presumed that *TMPRSS6* mutations result in elevated HFE2 protein levels at the hepatocyte plasma membrane, leading to enhanced signaling through the BMP/HFE2/SMAD signal transduction pathway and, consequently, elevated expression of hepcidin [9,12].

The proposed scheme implies that TMPRSS6 could function as an important regulatory protein which determines hepcidin expression by controlled cleavage of hepatocyte HFE2. Since it is very well known that mouse liver hepcidin (*Hamp*) mRNA content sensitively reacts to iron deficiency, iron overload or the rate of erythropoiesis [7], it is essential to know how TMPRSS6 protein content changes in response to these stimuli. It has already been reported that, in rats, TMPRSS6 protein increases following short-term administration of iron-deficient diet [13], which supports the role of TMPRSS6 in hepcidin downregulation. In addition, *in vitro* studies demonstrated an increase in TMPRSS6 protein or *Tmprss6* mRNA following hypoxia [14,15], again in line with the proposed function of TMPRSS6 as a negative regulator of hepcidin expression. On the contrary, iron administration to mice has been reported to increase *Tmprss6* mRNA content [16], while administration of iron to rats, albeit at a relatively low dose, did not influence liver TMPRSS6 protein [13]. Overall, the *in vivo* regulation of TMPRSS6 has not yet been fully elucidated.

The aim of this study was to determine TMPRSS6 protein content in experimental animals subjected to iron deficiency, iron overload or erythropoietin (EPO) administration. In addition, TMPRSS6 protein content was determined in HFE2-deficient mice. The results demonstrate that all these stimuli influence TMPRSS6 protein content, generally supporting the important role of TMPRSS6 in hepcidin gene regulation. Data from HFE2-deficient mice suggest that HFE2 enhances TMPRSS6 protein stability, supporting the concept of an interaction between these two proteins.

## Materials and Methods

### Animals and Treatment

All animal experiments were approved by the Czech Ministry of Education, protocol MSMT-1461/2015-5. Efforts were made to limit animal suffering: Intraperitoneal injections were performed under ether anesthesia, animals were sacrificed by decapitation under ether anesthesia.

Female outbred Wistar rats (170–195 g, Anlab SRO, Prague, Czech Republic) and male C57BL/6 mice (25–30 g) received four daily doses of EPO (NeoRecormon, Roche, 500 IU/day and 50 IU/day respectively) and were sacrificed 24 hours after the last injection. For iron-deficiency experiments, young female Wistar rats (45–55 g) or weaned four week old male C57BL/6 mice (14–17 g) were placed on an iron deficient diet for three or four weeks respectively. For iron overload experiments, iron was administered as iron dextran (Sigma Aldrich) to male adult C57BL/6 mice at 200, 350 and 750 mg/kg by single intraperitoneal injection; animals were sacrificed one week after treatment. For short-term iron overload experiments, animals were administered a single intraperitoneal injection of iron dextran at 1000 mg/kg and were sacrificed 24 h later [17].

Female mice with disruption of the *Hfe2* gene coding for hemojuvelin (*Hfe2*-/- mice), age 3–4 months, were originally a generous gift from Prof. Silvia Arber, Basel, Switzerland [18]. Female *Bmp6*-null mice were a generous gift from Prof. Roel Goldschmeding, University Medical Center Utrecht, the Netherlands [19]. The EPO administration scheme for *Hfe2*- and *Bmp6*-null mice was identical to C57BL/6 mice. Liver samples from *mask* mice [2], lacking the proteolytic domain of TMPRSS6, were a generous gift from Dr. Pauline Lee and Dr. Xin Du, Scripps Research Institute, La Jolla, CA, USA.

### Sample Preparation for Immunoblotting

For all experiments, a plasma membrane-enriched fraction obtained by centrifugation at 3000 g was used. Samples of liver (approx. 250 mg) were homogenized with a 6 mm Ultra Turrax homogenizer (3 x 10 s at maximum speed) in 2 ml of 10 mM HEPES, pH 7.4, containing protease inhibitors and 2 mM EDTA. The homogenate was centrifuged for 10 min at 400 g, and the supernatant was centrifuged at 3000 g for 15 min. The pellet was resuspended (Ultra Turrax, 10 s) in 10 mM HEPES containing 2 M NaCl to deplete the sample of co-precipitated non-membrane proteins associated by ionic bonds [20] and recentrifuged at 3000 g for 15 min. Subsequently, the pellet was resuspended in 0.1 M sodium carbonate, agitated for 1 h at 4°C and recentrifuged. This step is postulated to remove soluble proteins trapped in vesicles formed during homogenization [20]. Next, the pellet was resuspended in 10 mM HEPES containing 4 M urea and 100 mM NaCl to wash out residual non-membrane proteins. Finally, the pellet was washed with 10 mM HEPES and resuspended by sonication in 125 μl of 2% SDS containing 25 mM of ammonium bicarbonate. Insoluble material was pelleted by centrifugation at 16 000 g for 10 min and the supernatant was aliquoted and stored at -80°C. Typical yield for the washed 3000 g fraction was about 1.5 mg of protein per 1 g of liver. Immunoblotting was performed under reducing conditions as previously described [21]. Primary antibodies were: Rabbit anti-TMPRSS6, Ab56182, Abcam, 1:750; goat anti-HFE2, AF 3634, R&D Systems, 1:1500; goat anti-neogenin, AF1079, R&D Systems, 1:1000, and rabbit anti- Na+/K+ATPase α, SC-28800 (ATP1A), Santa Cruz, 1:10 000. Secondary antibodies (anti-rabbit, 711-036-152, 1:40 000 and anti-goat, 705-036-147, 1:40 000) were from Jackson Immunoresearch.

### Real-Time PCR, PNGase F Treatment, and Iron Determinations

Real-time PCR was performed on a Biorad IQ5 instrument using SYBR Green protocol, primers are listed in S1 Table. For the removal of N-linked oligosaccharides, PNGase F (New England Biolabs) was used according to manufacturers instructions. Iron was determined according to Torrance and Bothwell [22].

### Statistical Analysis

For densitometric quantification, TMPRSS6 band densities obtained on a Biorad GS-800 densitometer were normalized to their respective loading control band densities; treated *vs*. control sample values were then compared using paired t-test. Graphed band density data, generally calculated from several blots, are expressed in % of control, control values were set as 100%. For small data sets (n = 3), values obtained from single 15-well gels were analyzed by unpaired t-test. PCR data were evaluated by the Mann-Whitney test. Data are expressed as means ± SD.

## Results

### HFE2 and TMPRSS6 are Present as Full Length Proteins in the Liver 3000 g Fraction

**Fig 1:**
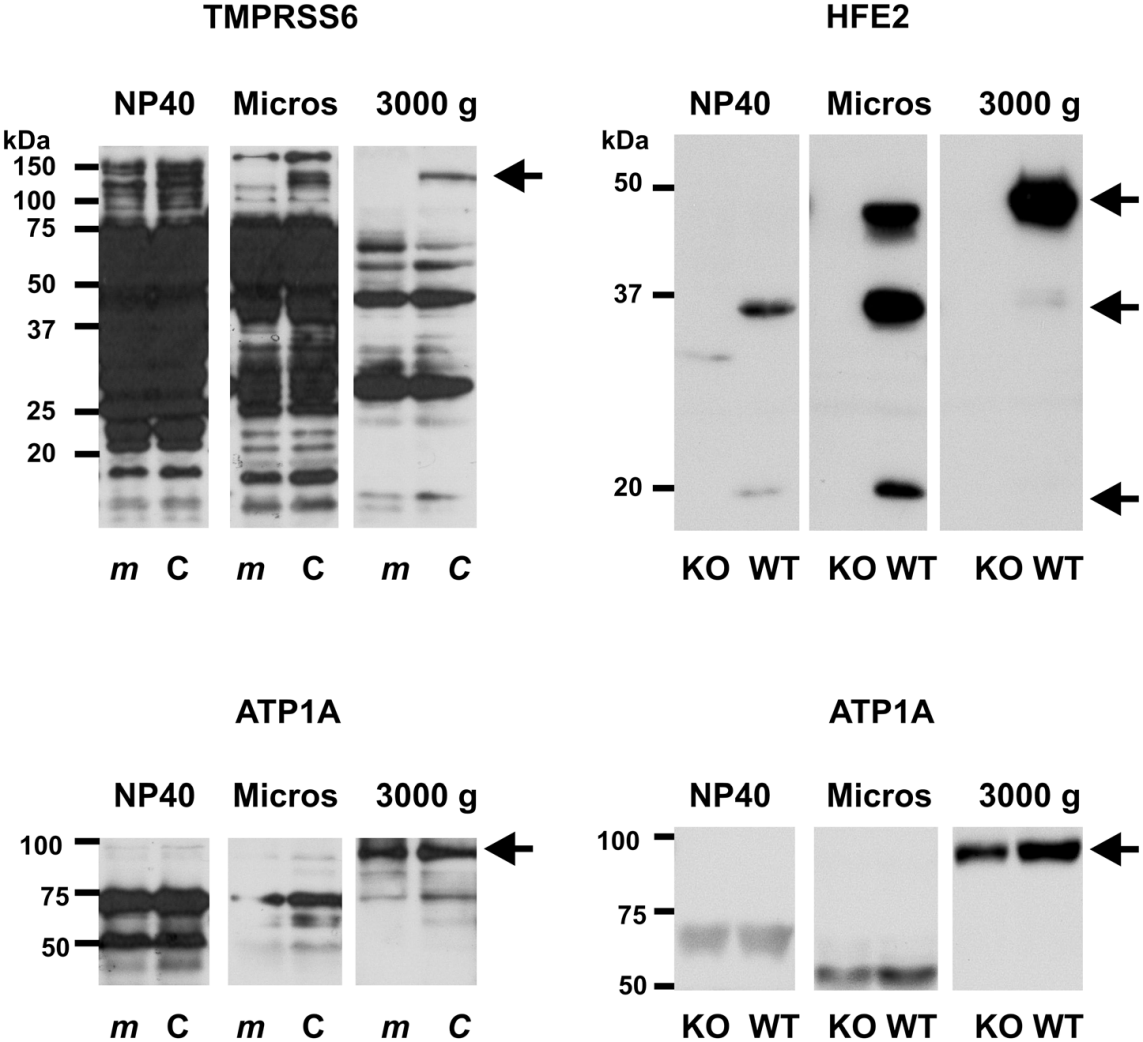
Immunoblot of TMPRSS6, HFE2 and ATP1A in the 3000 g fraction. Detection of TMPRSS6, HFE2 and ATP1A is enhanced in the 3000g fraction of mouse liver homogenates (3000g) in comparison with whole liver homogenates prepared in 1% NP40 (NP40) or microsomes (Micros). Samples were prepared from C57BL/6 mice (C), *mask* mice (*m*), *Hfe2*+/+ mice (WT) and *Hfe2*-/- mice (KO). Arrows denote the TMPRSS6-, HFE2- and ATP1A-specific bands. In contrast to the NP40 whole liver homogenate or liver microsomes, HFE2 is present in the 3000 g fraction solely as a full-length protein.

HFE2 has been clearly identified as a TMPRSS6 substrate in *in vitro* studies [9]. In order to examine the interaction between TMPRSS6 and HFE2 *in vivo*, it was necessary to select a cellular fraction which would allow reproducible quantification of both proteins. In our previous experiments, we found that HFE2 is easier to detect in microsomal fraction than in whole homogenate [23]. However, published studies on HFE2 processing indicate that HFE2 undergoes complex retrograde trafficking to the Golgi compartment [24]. As liver microsomes also contain parts of Golgi membranes, while the proposed cleavage of HFE2 occurs at the plasma membrane, liver microsomes do not appear to be ideally suited for the investigation of the TMPRSS6/HFE2 interaction. Therefore, a partially purified membrane fraction obtained by centrifugation at 3000 g was used for the current studies. The detection of TMPRSS6, and particularly the detection of HFE2, was significantly improved in this fraction, as was the detection of ATP1A, a marker of the plasma membrane (Fig 1). Whereas TMPRSS6 was detected as a 120 kDa band in all samples, the size of the HFE2 band was influenced by the method of sample preparation and the detergent used. In our previous studies using NP-40-containing liver homogenates, as well as in detergent-free microsomes, HFE2 was detected as a heterodimer composed of two bands of 35 and 18 kDa [21,23]; in the present study using the 3000 g fraction HFE2 was present as a strong band of approximately 52 kDa (Fig 1). This size corresponds to full-length, glycosylated HFE2 protein. These data suggest that whereas the major part of cellular HFE2 protein exists as a heterodimer, a small amount of the protein is present at the plasma membrane as the uncleaved full-length 52 kDa chain.

*In vivo* studies on TMPRSS6 protein have so far used mostly non-commercial antibodies [13,25]. In an attempt to identify a suitable commercially available antibody, we used liver samples from C57BL/6 and *mask* mice. The Ab56182 antibody, raised against the catalytic domain of TMPRSS6, enabled detection of a TMPRSS6-specific band of about 120 kDa which was not present in samples from *mask* mice (Fig 1). The observed size corresponds to reported TMPRSS6 size in rats and mice [13,25], and very probably represents the full-length mouse protein (811 amino acids, predicted molecular weight 91 kDa plus 7 glycosylations). On mouse liver blots, an additional lower band of approximately 110 kDa was occasionally seen; however, since this band was also weakly detectable in some *mask* mice samples, only the 120 kDa band was used for quantification.

TMPRSS6 is reported to undergo proteolytic activation which is proposed to produce a non-glycosylated catalytic domain fragment of about 26 kDa, connected via disulfide bridges to the membrane-bound part of the protein [26,27]. In agreement with published data [13,28], no TMPRSS6-specific band of this size was observed on immunoblots (Fig 1), suggesting that the amount of activated TMPRSS6 is small compared to the full-length protein.

### TMPRSS6 Protein is Increased in Iron-deficient Rats and Mice

It has previously been reported that TMPRSS6 increases following short-term administration of iron-deficient diet to weaned rats [13]. For our experiments, we used young mice and rats kept on an iron-deficient diet for several weeks. The treatments resulted in iron-deficiency anemia and a significant decrease of liver iron concentration (Table 1).

**Table 1:** Iron content in iron-deficient and EPO-treated experimental animals.

Group and Treatment	Hemoglobin	Hematocrit	Liver Iron
	g/litre	%	μg/g wet wt.
Mouse, control	141±12	40±3	46±13
Mouse, iron deficiency	110±8*	37±2	20±2*
Mouse, EPO	162±17*	48±4*	33±9*
Rat, control	140±1	42±1	157±12
Rat, iron deficiency	53±5*	17±3*	19±2*
Rat, EPO	161±5*	53±3*	111±26*
*Tmprss6* +/+ mouse	140±8	44±4	66±5
*Mask* mouse	104±6*	37±3	21±3*

Data are expressed as mean ± SD, n = 5 for male mice, n = 4 for female rats and n = 3 for male *mask* mice. Asterisk denotes statistical significance compared to controls (p<0.05).

**Fig 2:**
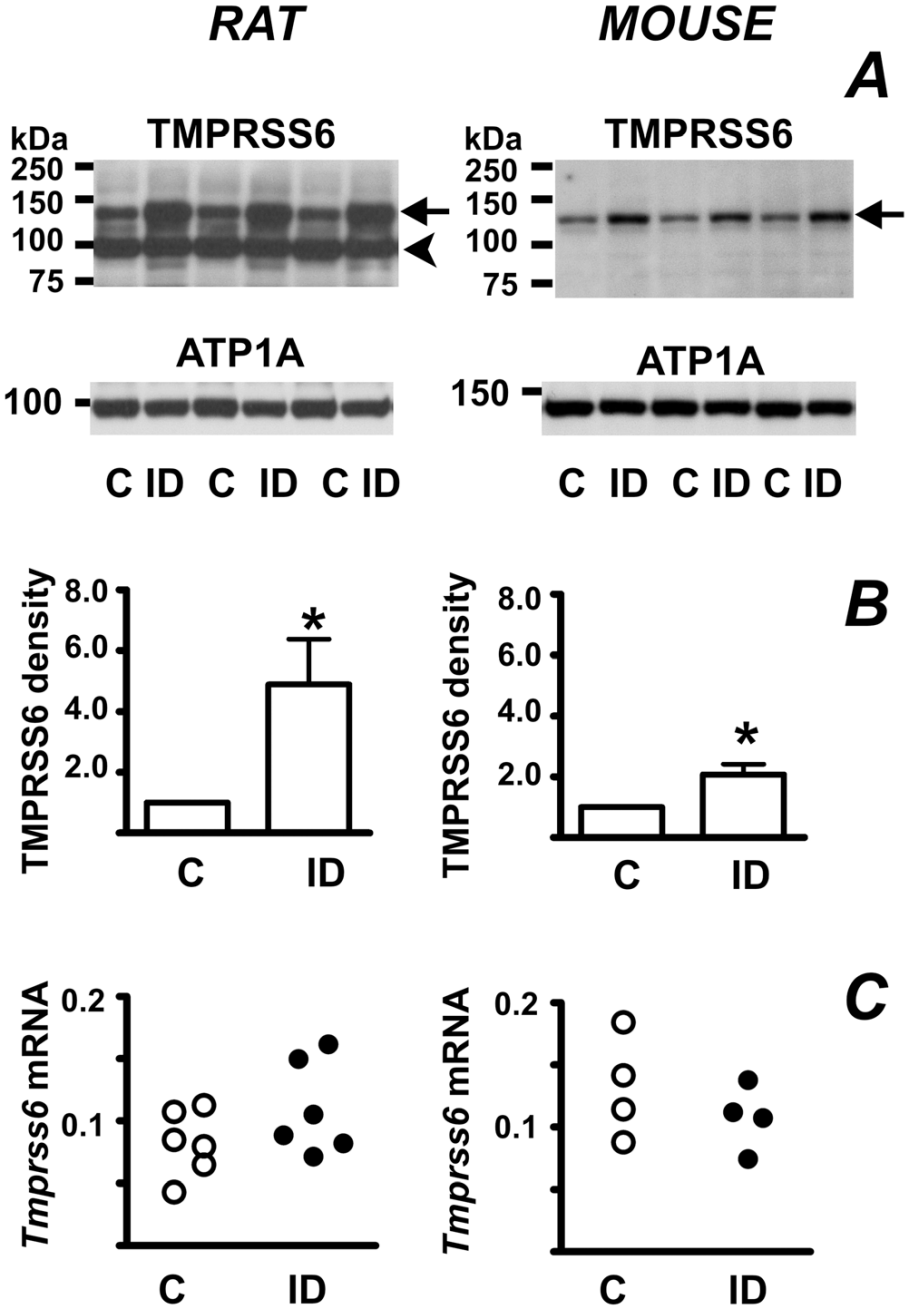
Iron deficiency increases TMPRSS6 protein content. (A) Increase of TMPRSS6 protein content in rats and mice kept on an iron-deficient diet. Control animals (C) were fed standard laboratory diet, iron-deficient animals (ID) were fed an iron-deficient diet since weaning. Arrows denote TMPRSS6-specific bands, arrowhead denotes a non-specific band present in rat liver samples. ATP1A is used as loading control. (B) TMPRSS6 band density quantifications. n = 4, asterisk denotes statistical significance (p<0.05). (C) Effect of iron deficiency anemia on liver *Tmprss6* mRNA content. *Tmprss6* mRNA content is determined relative to *Actb* mRNA.

The 120 kDa TMPRSS6-specific band was significantly increased in both mice and rats (Fig 2A and 2B). Liver *Tmprss6* mRNA content tended to increase in iron-deficient rats, but the changes did not reach statistical significance (Fig 2C). Rat liver samples probed with the Ab56182 antibody displayed, in addition to the 120 kDa TMPRSS6 band, a relatively strong band at 100 kDa (Fig 2A). Since this band did not react to any treatment, and was not seen in mouse samples, we regard it as a nonspecific band.

### TMPRSS6 Protein is Increased in Erythropoietin-treated Rats and Mice

**Fig 3:**
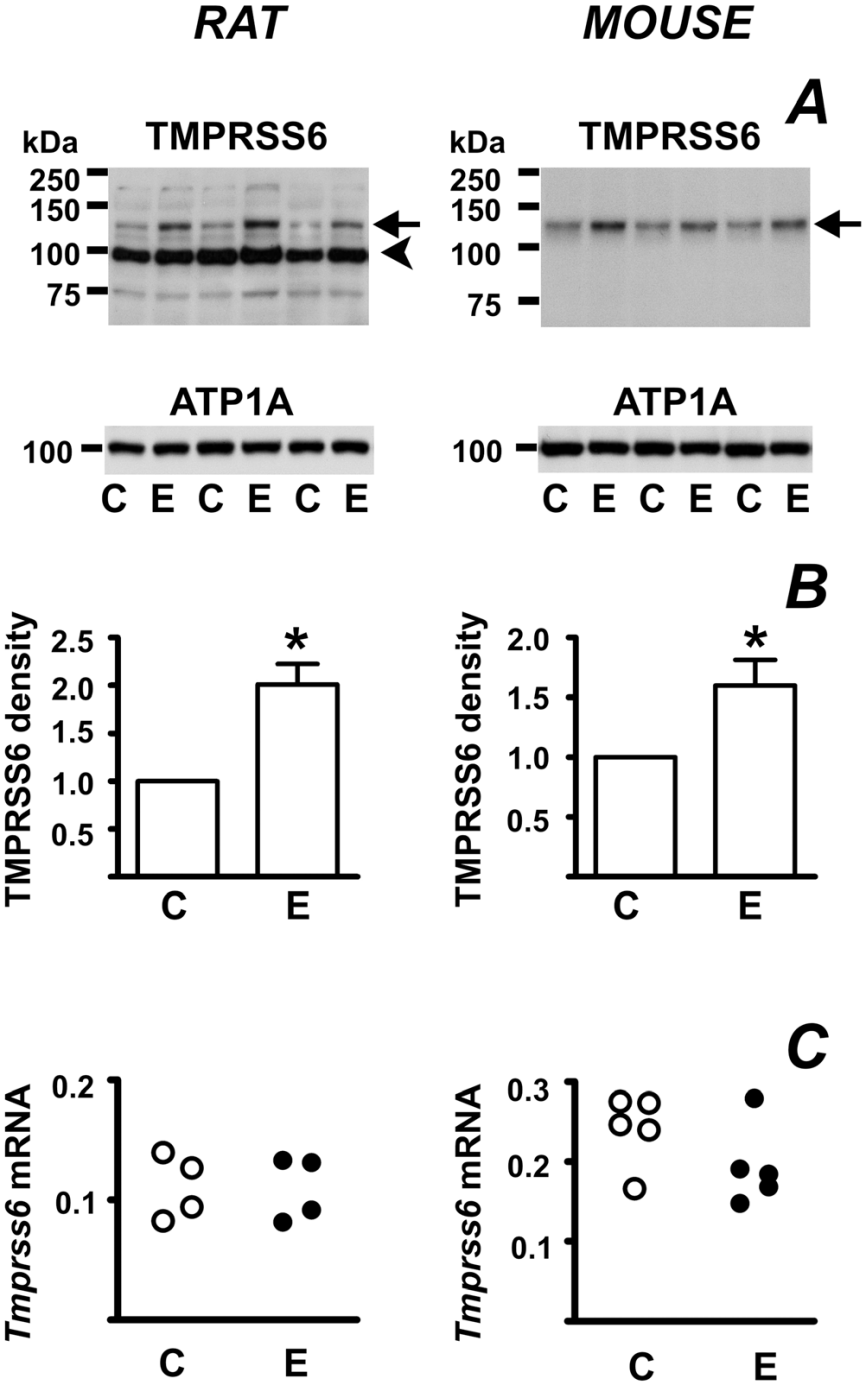
Erythropoietin treatment increases TMPRSS6 protein content. (A) Increase of TMPRSS6 protein content in rats and mice treated with saline (C) or EPO (E) at 500 or 50 IU/animal for four days. Arrows denote TMPRSS6-specific bands, arrowhead denotes a nonspecific band present in rat liver samples. ATP1A is used as loading control. (B) TMPRSS6 band density quantification. Asterisk denotes statistical significance (p<0.05), n = 5 for rats and n = 6 for mice. (C) Lack of effect of EPO treatment on *Tmprss6* mRNA. *Tmprss6* mRNA content is determined relative to *Actb* mRNA.

Hepcidin expression is strongly affected by the rate of erythropoiesis [7]. Therefore, it was of interest to determine the effect of EPO administration on TMPRSS6 protein expression. As expected, administration of high doses of EPO for four days resulted in dramatic downregulation of *Hamp* mRNA (less than 0.1% of control values, S1 Fig). TMPRSS6 protein content increased in both rats and mice (Fig 3A and 3B); the effect in rats was more pronounced. The effect of EPO on TMPRSS6 protein was posttranscriptional, as *Tmprss6* mRNA content was not influenced by EPO treatment (Fig 3C).

Since the administration of EPO for four days resulted in a significant increase of blood hemoglobin and a decrease of liver iron (Table 1), it was not possible to determine whether the effect of EPO on TMPRSS6 protein content was primarily related to accelerated erythropoiesis, or to decreased iron availability. Therefore, we performed an additional experiment, in which rats were sacrificed 24 hours after EPO administration. In this experimental setting, liver *Hamp* mRNA content decreased only to approximately 25% of controls and liver iron content was not affected (S2 Fig). Liver TMPRSS6 protein content was not significantly changed (S2 Fig), indicating that EPO influences TMPRSS6 protein content only after prolonged administration, which also substantially affects iron homeostasis.

### Regulation of TMPRSS6 Protein by Erythropoietin is Intact in *Bmp6*-Mutant Mice

**Fig 4:**
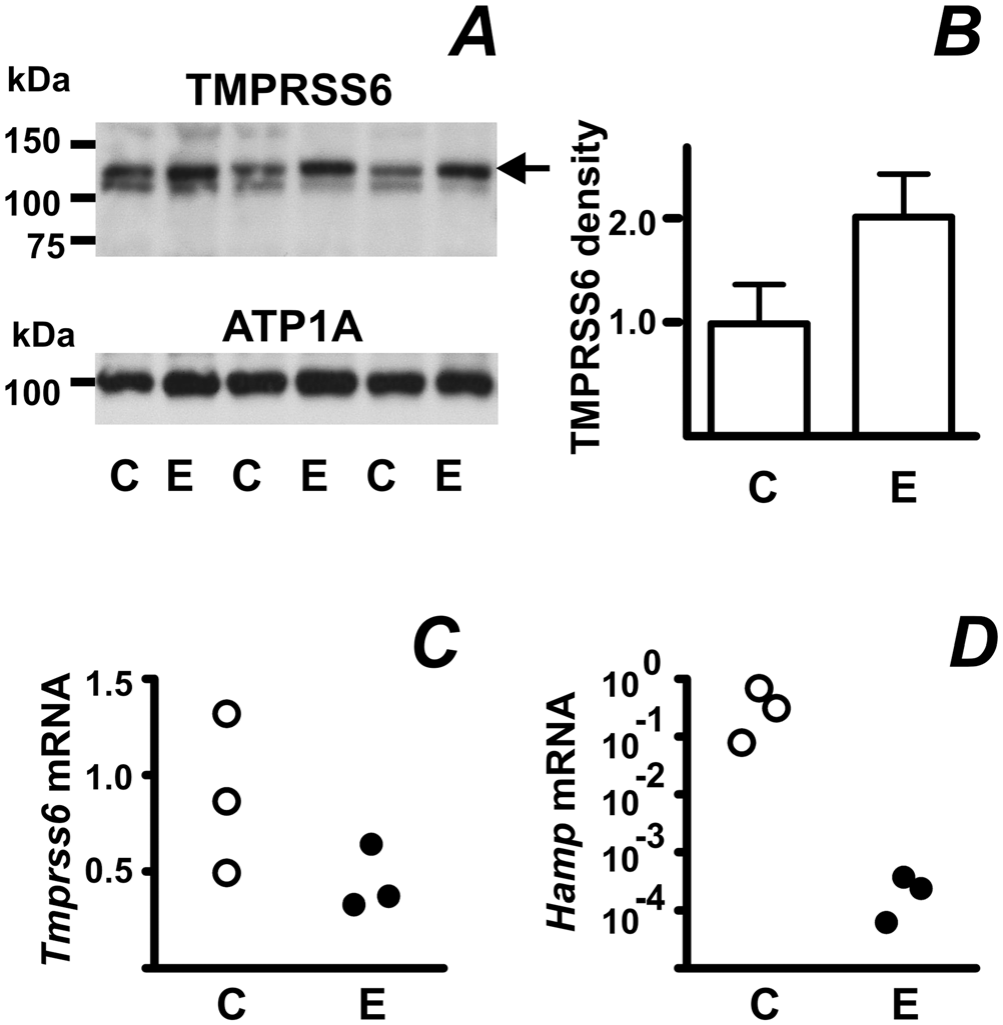
Erythropoietin treatment increases TMPRSS6 protein content in *Bmp6*-mutant mice. (A) Increase of TMPRSS6 protein content in *Bmp6*-mutant mice mice treated with saline (C) or EPO (E) at 50 I.U./mouse for four days. Arrow denotes TMPRSS6-specific band. ATP1A is used as loading control. (B) TMPRSS6 band density quantification. (C and D) Effect of EPO treatment on *Tmprss6* and *Hamp* mRNA content. *Tmprss6* and *Hamp* mRNA content is determined relative to *Actb* mRNA.

BMP6 protein is regarded as a key extracellular molecule which initiates iron-dependent signaling at the hepatocyte plasma membrane through the BMP6/HFE2/SMAD pathway, ultimately leading to an increase in hepcidin transcription [29,30]. It was therefore of interest to determine whether the observed regulation of TMPRSS6 protein by EPO remains functional in *Bmp6*-null mice. In accordance with results obtained in C57BL/6 mice, EPO tended to increase TMPRSS6 protein content in *Bmp6*-null mice (Fig 4A and 4B) by a posttranscriptional mechanism (Fig 4C). Although the results did not reach statistical significance, due to limited number of available mouse pairs (n = 3), they nevertheless indicate that the EPO-mediated regulation of TMPRSS6 does not require functional BMP6 protein. Similarly to C57BL/6 mice, EPO treatment dramatically decreased *Hamp* mRNA in *Bmp6*-null mice (Fig 4D), demonstrating that BMP6 is dispensable for the regulation of *Hamp* expression by accelerated erythropoiesis.

### Iron Decreases Liver TMPRSS6 Protein Content in Rats and Mice

**Fig 5:**
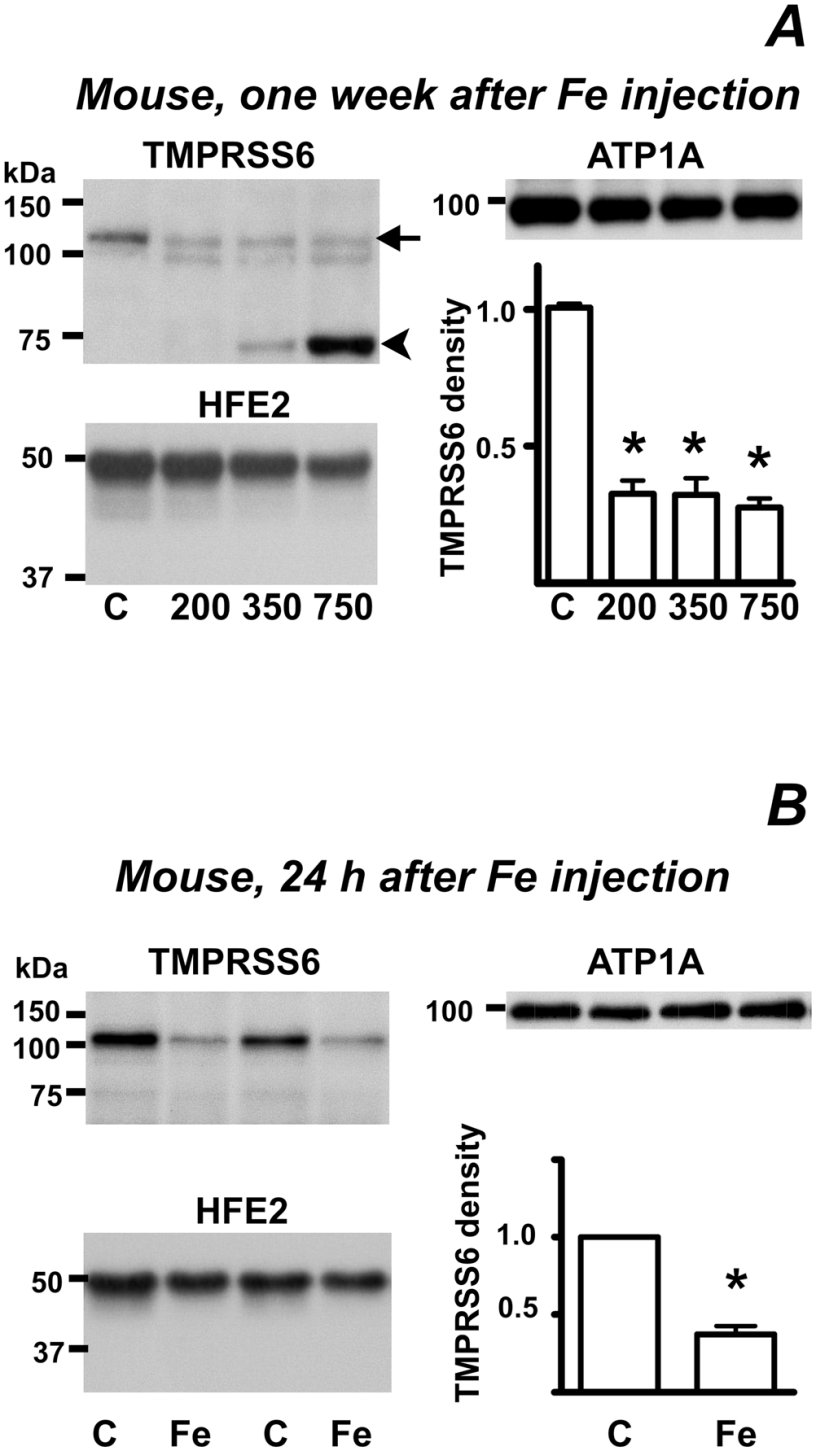
High dose of iron dextran decreases TMPRSS6 protein content. (A) Effect of iron on TMPRSS6 and HFE2 protein content. Mice were treated with 200, 350 and 750 mg/kg of iron dextran one week before sacrifice, control mice (C) received physiological saline. ATP1A is used as loading control. Arrow denotes the TMPRSS6-specific protein band, arrowhead denotes a nonspecific band. Graph represents TMPRSS6 band density quantification, asterisk denotes statistical significance (p<0.05, n = 3). (B) Effect of a 1000 mg/kg of iron dextran administered to mice 24 h prior to sacrifice. Graph represents TMPRSS6 band density quantification, asterisk denotes statistical significance (p<0.05, n = 4).

Injection of iron dextran at 200, 350 and 700 mg/kg decreased TMPRSS6 protein (Fig 5A). However, lower doses of 50 and 100 mg/kg did not produce an effect (S3 Fig). As expected [7,10], iron treatment increased *Hamp* and *Bmp6* expression (S4 Fig). In contrast to the decrease of TMPRSS6 protein in mice treated with high doses of iron dextran (Fig 5A), *Tmprss6* mRNA content was slightly increased (S4 Fig). In livers from iron-treated mice, a strong additional dose-dependent band was apparent at 75 kDa (Fig 5A). Since this band did not react to PNGase F treatment, and was also seen in liver samples from *mask* mice kept on an iron-enriched diet (S5 Fig), we regard it as a non-specific band, probably related to the high intracellular liver iron content (S2 Table).

It has been reported that the response of *Hamp* gene to iron overload differs with different time intervals, and different sites of liver iron accumulation [17,31–33]. It was therefore of interest to determine whether the observed iron-induced decrease in TMPRSS6 protein content also occurs early after iron administration. To this purpose, we used mice injected with 1000 mg/kg of iron dextran 24 h prior to sacrifice. In this experimental setting, iron is reportedly not upregulating liver *Bmp6* expression [17], and the response of *Hamp* mRNA to iron remains limited (S4 Fig). TMPRSS6 protein content was again decreased (Fig 5B), suggesting that iron influences TMPRSS6 protein content and *Bmp6* gene expression by different pathways.

### Full Length HFE2 is Partially Cleaved in Iron-deficient Rats and Mice

**Fig 6:**
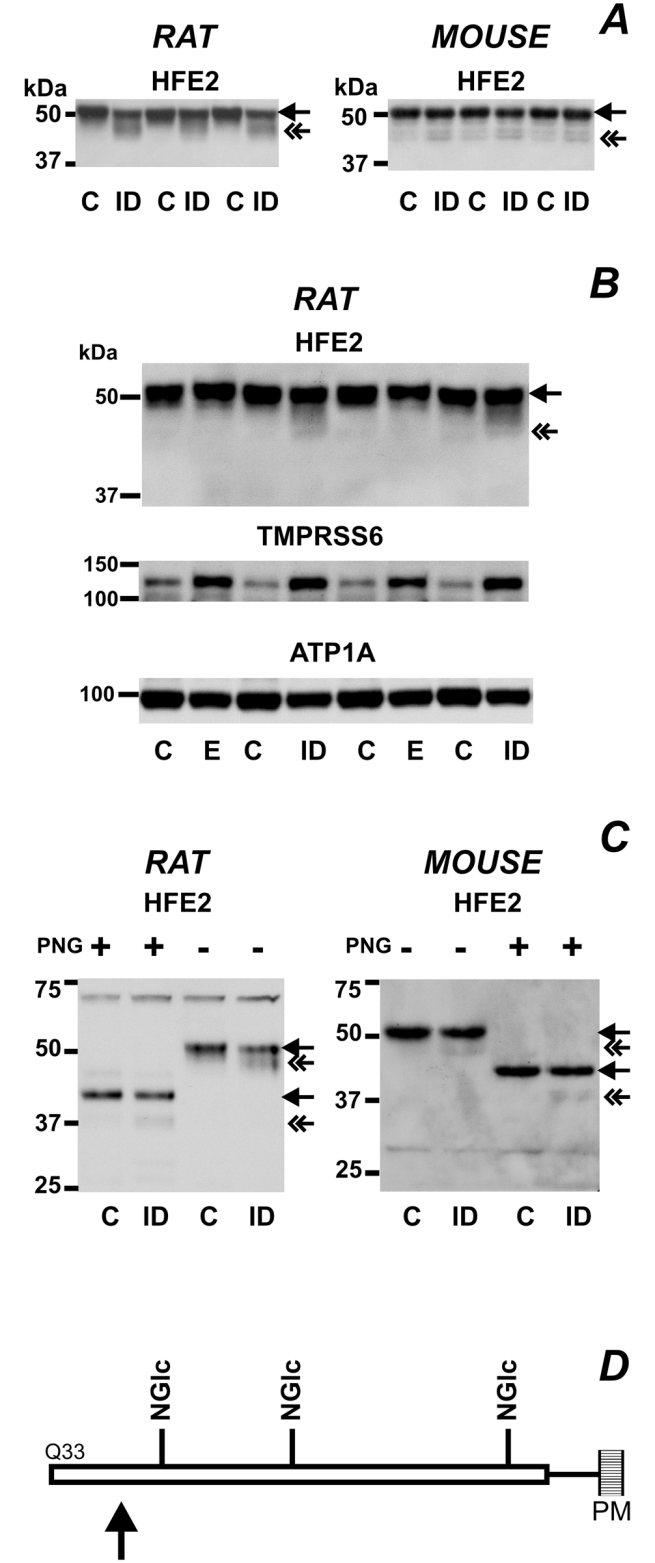
HFE2 is partially cleaved at the N-terminus in iron-deficient animals. (A) Partial cleavage of full length HFE2 (arrow) to an approximately 5 kDa shorter protein (double arrow) under iron deficiency conditions. Weaned rats and mice were kept on an iron deficient diet for three or four weeks respectively (ID), control animals (C) received standard laboratory diet. Loading controls are similar to Fig 2A. (B) Comparison of the effects of iron-deficient diet (ID) or EPO treatment (E) on TMPRSS6 protein content and HFE2 cleavage in rats. Both treatments increase TMPRSS6 protein content; cleaved HFE2 band (double arrow) is apparent only in iron-deficient animals. (C) Cleaved HFE2 protein has the same number of N-linked oligosaccharides as the full length protein. Samples from control (C) or iron-deficient (ID) rats and mice were incubated with PNGase F (PNG +) or water (-). PNGase F treatment reduced the size of full length HFE2 protein (arrow) by about 10 kDa; the same size reduction of approximately 10 kDa was observed for the cleaved HFE2 protein (double arrow) present in iron-deficient samples. (D) Schematic representation of full length mouse HFE2 protein. The 361 amino acid chain has Q33 as the N-terninal amino acid (UniProt entry Q7TQ32), and is bound with a GPI anchor to the plasma membrane (PM). Arrow denotes the approximate position of the observed cleavage site, which is located N-terminally of the three N-linked oligosaccharide (NGlc) chains.

In previous studies using liver microsomes, we found unchanged HFE2 protein content in samples from iron-deficient mice [34]. However, in the present study, the 3000 g samples from iron-deficient animals displayed, in addition to the main band of approximately 52 kDa, an additional weak truncated band of approximately 47 kDa (Fig 6A). Since TMPRSS6 protein was increased in the same samples from iron-deficient rats and mice (Fig 2A), it is theoretically possible that this shorter band represents a TMPRSS6-cleaved fragment of full-length HFE2. In contrast to the effect of iron deficiency, EPO treatment did not result in increased cleavage of HFE2, despite an increase in TMPRSS6 protein content (Fig 6B). These results possibly indicate that increased cleavage of HFE2 requires prolonged treatment and/or severe iron deficiency.

The modest shift of the cleaved band *vs*. the full-length protein (approximately 4 to 6 kDa) indicates a possible cleavage site at one of the several arginines at the N-terminus of HFE2. To determine whether the cleavage occurs in the glycosylated part of the protein, low-iron samples displaying distinct cleaved bands were treated with PNGase F, which removes N-linked oligosaccharides. This treatment reduced the apparent mass of the full length HFE2 protein by approximately 10 kDa (from 52 to 42 kDa); the size of the cleaved band present in iron-deficient samples was reduced by PNGase F treatment from 47 kDa to approximately 37 kDa (Fig 6C). These data demonstrate that the iron deficiency-induced cleavage of HFE2 does not affect the number of N-linked oligosaccharides present in the membrane-bound part of the protein, indicating that the cleavage site is located N-terminally to the first N-linked oligosaccharide (Fig 6D).

### TMPRSS6 Protein is Decreased in *Hfe2*-/- Mice

**Fig 7:**
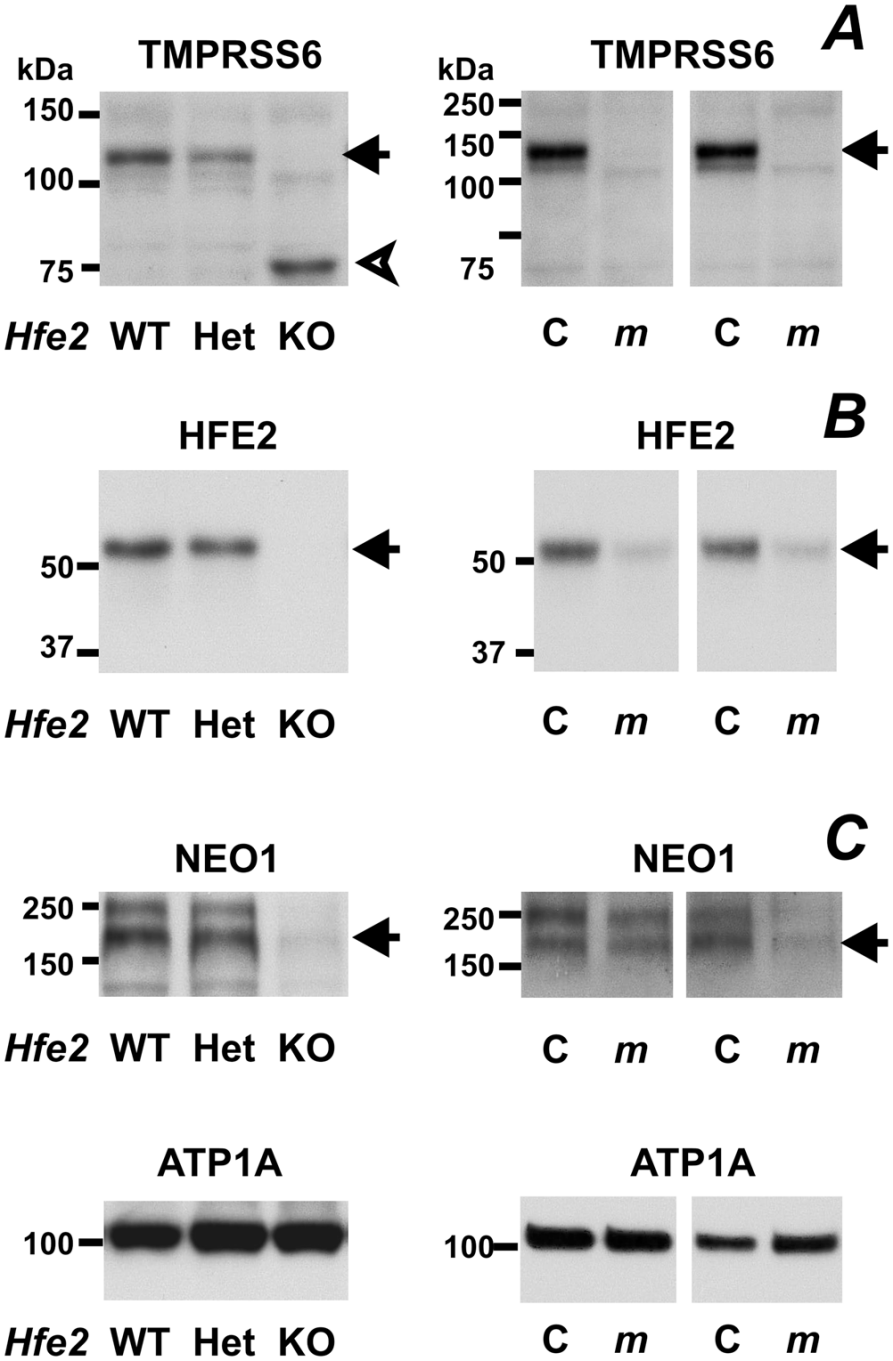
TMPRSS6 protein content and neogenin protein content is decreased in *Hfe2-/-* mice. (A) Immunoblot of TMPRSS6 protein in the liver 3000 g fraction of *Hfe2*+/+, (*Hfe2* WT), *Hfe2*-/+ (Het), *Hfe2*-/- (KO), C57BL/6 (WT) and *mask* (*m*) mice. Arrow denotes the TMPRSS6-specific band; arrowhead indicates a nonspecific band. *Mask* mice samples represent results from two experiments with two pairs of mice. (B) and (C) Immunoblots of HFE2, neogenin (NEO1) and ATP1A (loading control) in the same samples. Arrows denote HFE2- and NEO1-specific bands.

HFE2 is the proposed target for the protease activity of TMPRSS6 [9]. However, in our previous studies with whole liver homogenates and liver microsomes [21], we found decreased, rather than increased, HFE2 protein content in *mask* mice. A similar decrease in full-length HFE2 protein in *mask* mice was confirmed in the present study utilizing the 3000 g membrane fraction (Fig 7B). *Vice versa*, the amount of TMPRSS6 protein was strongly decreased in liver samples from *Hfe2*-/- mice (Fig 7A). In addition, *Hfe2*-/- mice displayed strongly decreased content of neogenin (Fig 7C), another protein reportedly participating in *Hamp* gene regulation [25,35].

Although *Hfe2*-/- and *Bmp6*-/- mice are almost identical with respect to the iron overload phenotype [18,29,30], their liver TMPRSS6 protein content is markedly different, with *Hfe2*-/- mice displaying almost no detectable full length TMPRSS6 protein (Fig 7A) and no response of TMPRSS6 to EPO treatment (S6 Fig). In contrast, TMPRSS6 was readily detectable in *Bmp6*-/- mice (Fig 4A).

Similar to mice injected with iron, the anti-TMPRSS6 antibody detected a strong band at 75 kDa in *Hfe2*-/- mice (Fig 7A). As the position of this band did not change following PNGase F treatment (S5 Fig), it very probably represents a non-specific band.

## Discussion

TMPRSS6 is a hepatocyte transmembrane serine protease whose mutations result in iron-refractory iron deficiency anemia [1]. The identification of TMPRSS6 as a potent negative regulator of hepcidin, the key iron-regulatory hormone, has raised the possibility that hepcidin expression could be regulated by modulation of TMPRSS6-dependent proteolytic activity. However, very little information is available on the *in vivo* regulation of TMPRSS6 protein levels.

Hepcidin expression is known to respond to iron overload and iron deficiency, as well as to the rate of erythropoiesis [7]. Therefore, the primary aim of this study was to determine TMPRSS6 protein content in plasma membrane-enriched fractions of liver samples from experimental animals subjected to manipulations of iron homeostasis or EPO administration. Since HFE2 is the proposed target of TMPRSS6, HFE2 protein levels were determined as well.

Analysis of samples from iron-deficient animals confirmed the previously reported [13] increase of TMPRSS6 protein by iron deficiency. *Tmprss6* mRNA did not significantly change in iron deficiency anemia, highlighting the importance of posttranscriptional regulation of TMPRSS6 [36]. Interestingly, immunoblotting of HFE2 protein indicated that in iron-deficient animals the full length HFE2 protein is partially cleaved. Since TMPRSS6 protein content was increased in the same samples, these results indirectly support the proposed concept that membrane HFE2 could be the physiological target of TMPRSS6 [9]. The modest band shift of about 3–6 kDa suggests that HFE2 is cleaved relatively close to the N-terminus, while treatment with PNGase F indicates that the resulting shortened membrane-bound fragment retains all three of its N-linked oligosaccharides (Fig 6D). In this respect, it is interesting to note that the ligand of HFE2, BMP6, was proposed to bind at the N-terminal part of HFE2 [37]. It is therefore possible that the partial cleavage of HFE2 at the N-terminus could diminish its ability to function as BMP6 coreceptor. In conditions of iron deficiency, increased TMPRSS6 protein content could thus result in decreased activity of the BMP6/HFE2 signaling pathway, contributing to the well-documented decrease of *Hamp* expression.

In sufficiently exposed blots, the cleaved band could also be seen in control samples, demonstrating that HFE2 cleavage occurs to some degree in control livers as well. However, even under conditions of severe iron deficiency, the intensity of the truncated band was much weaker than the intensity of the full-length protein band, suggesting that only a minor part of the full-lengh plasma membrane HFE2 is actually cleaved.

Injections of iron dextran to mice decreased liver TMPRSS6 protein content by a posttranscriptional mechanism. This response of TMPRSS6 to iron again confirms the inverse correlation between hepatic *Hamp* mRNA content and TMPRSS6 protein content, indicating that the modulation of TMPRSS6 activity could be an important factor in the regulation of hepcidin expression.

Clinical as well as experimental studies indicate that TMPRSS6 is particularly important for the appropriate downregulation of hepcidin expression by signals related to accelerated erythropoiesis. It has for a long time been known that administration of EPO to experimental animals downregulates *Hamp* transcription [38]. This EPO-mediated decrease of *Hamp* expression occurs not only in rats and wild-type mice, but also in HFE2-deficient mice [39,40], TFR2-deficient mice [40], and BMP6-deficient mice (Fig 4D). In striking contrast to these animals, TMPRSS6-deficient mice fail to decrease *Hamp* mRNA content following EPO treatment [41,42]. In addition, it has been reported that EPO administration does not improve anemia in patients with *TMPRSS6* mutations [43]. These reports strongly suggest that TMPRSS6 is a crucial component of the “erythroid regulator” which increases iron absorption in response to erythropoietic demand [44]. In agreement with this concept [45,46], we found increased TMPRSS6 protein content in the livers of EPO-treated mice and rats. Intriguingly, although liver TMPRSS6 protein increased both in response to EPO treatment or in response to iron deficiency, the partial cleavage of HFE2 protein was not observed in EPO-treated animals, possibly indicating that detectable cleavage occurs only in conditions of prolonged and severe iron depletion.

The exact pathway(s) which decrease *Hamp* expression following administration of EPO are at present under intensive investigation. Very probably, the dramatic downregulation of liver *Hamp* mRNA content observed in EPO-treated experimental animals results from a combination of two main events: The EPO-induced decrease in liver and plasma iron content, which causes decreased signaling through the iron-sensing signaling pathways, and the synthesis, in the activated bone marrow and spleen, of specific factor(s) mediating *Hamp* downregulation by an yet unknown mechanism [40,47]. It is not clear whether the observed EPO-induced increase in TMPRSS6 protein is related solely to the EPO-induced efflux of liver and plasma iron into the erythron, as suggested by the observed lack of effect of short-term EPO administration, or whether EPO also increases TMPRSS6 protein content by some other mechanism. In any case, the increase of TMPRSS6 protein content observed in mice and rats treated with EPO for several days could, by downregulating BMP6/HFE2 signaling, contribute to the profound downregulation of *Hamp* expression in these animals.

Although HFE2 is the most probable target of TMPRSS6, we previously paradoxically found decreased, rather than increased, amount of the autocatalytically cleaved 35 kDa HFE2 fragments in microsomes from *TMPRSS6*-mutated mice [21,23]. In the present study, we confirm decreased HFE2 protein content in the liver 3000 g fraction from *mask* mice. To further study the interaction between TMPRSS6 and HFE2, we now reciprocally determined TMPRSS6 protein levels in *Hfe2*-/- mice (Fig 7A). Interestingly, disruption of the *Hfe2* gene resulted in a dramatic decrease of TMPRSS6 protein content. A possible explanation for this observation is that TMPRSS6 and HFE2, and possibly also neogenin, function as a protein complex in the hepatocyte plasma membrane, as previously reported by other groups [9,25]. Disruption of one component of this protein complex could then lead to accelerated degradation of the other component(s). This would explain the decreased liver TMPRSS6 and neogenin content in *Hfe2*-/- mice, as well as the decreased HFE2 content in *mask* mice.

Overall, the *in vivo* results reported in this study do support the reported [9] interaction between TMPRSS6 and HFE2 proteins. However, this does not exclude the possibility that, in addition to HFE2, TMPRSS6 could have other important physiological targets as well. While available literature data indicate that the presence of functional TMPRSS6 protein represents a *sine qua non* condition for the EPO-induced decrease of liver *Hamp* mRNA [41–43,45] it has also very recently been reported that EPO-mediated downregulation of liver *Hamp* expression does not occur in mice lacking the newly identified erythroblast-secreted factor erythroferrone [40]. This could indicate that both TMPRSS6 and erythroferrone are important components of one signaling pathway [46]. In this respect, it should be noted that despite the profound decrease in hepatic TMPRSS6 protein in *Hfe*2-/- mice (Fig 7A), these mice are still able to downregulate *Hamp* expression following EPO administration [39,40], possibly indicating that even a limited amount of Tmprss6 protein enables efficient erythroferrone signaling. Obviously, further studies will be necessary to elucidate the exact role of TMPRSS6, and to identify its possible additional substrates.

In conclusion, our study demonstrated, in both mice and rats, a posttranscriptional increase of liver TMPRSS6 protein by erythropoietin. In addition, the observed partial cleavage of HFE2 in liver samples with high TMPRSS6 protein content, as well as the substantial decrease of TMPRSS6 protein in *Hfe2*-/- mouse liver samples, strongly suggests that TMPRSS6 and HFE2 proteins interact at the hepatocyte plasma membrane.

## Supporting Information

S1 Fig Effect of iron deficiency and EPO administration on *Hamp* mRNA. (DOC)

S2 Fig Lack of effect of a single dose of EPO on TMPRSS6 protein content in rats. (DOC)

S3 Fig Lack of effect of low doses of iron-dextran on TMPRSS6 protein content in mice. (DOC)

S4 Fig Effect of iron on *Hamp*, *Bmp6* and *Tmprss6* RNA. (DOC)

S5 Fig The 75 kDa band observed in mouse liver samples with high iron content is not TMPRSS6-specific. (DOC)

S6 Fig Lack of effect of EPO on TMPRSS6 protein content in *Hfe2*-/- mice. (DOC)

S1 Table List of primers used for PCR analysis. (DOC)

S2 Table Liver iron content in iron-overloaded animals. (DOC)
