# Supplementary material for: Effect of Erythropoietin, Iron Deficiency and Iron Overload on Liver Matriptase-2 (TMPRSS6) Protein Content in Mice and Rats
Source: PLoS One. 2016 Feb 4;11(2):e0148540. doi: 10.1371/journal.pone.0148540 (PMC4742081; doi:10.1371/journal.pone.0148540)
Supplement: S1 Fig — (DOC) [file pone.0148540.s001.doc]

**S1 Fig. Effect of iron deficiency and EPO administration on *Hamp* mRNA.**

(A) *Hamp* mRNA content in liver samples from control rats (C) and rats kept for three weeks on a low-iron diet (ID).

(B) *Hamp* mRNA content in liver samples from control mice (C) and mice kept for four weeks on a low-iron diet (ID).

(C) *Hamp* mRNA content in liver samples from control rats (C) and rats administered four daily doses of EPO (E) at 500 IU/rat.

(D) *Hamp* mRNA content in liver samples from control mice (C) and mice administered four daily doses of EPO (E) at 50 IU/mouse.

*Hamp* mRNA content is expressed relative to *Actb* mRNA, asterisks denote statistical significance (*p*<0.05).

**
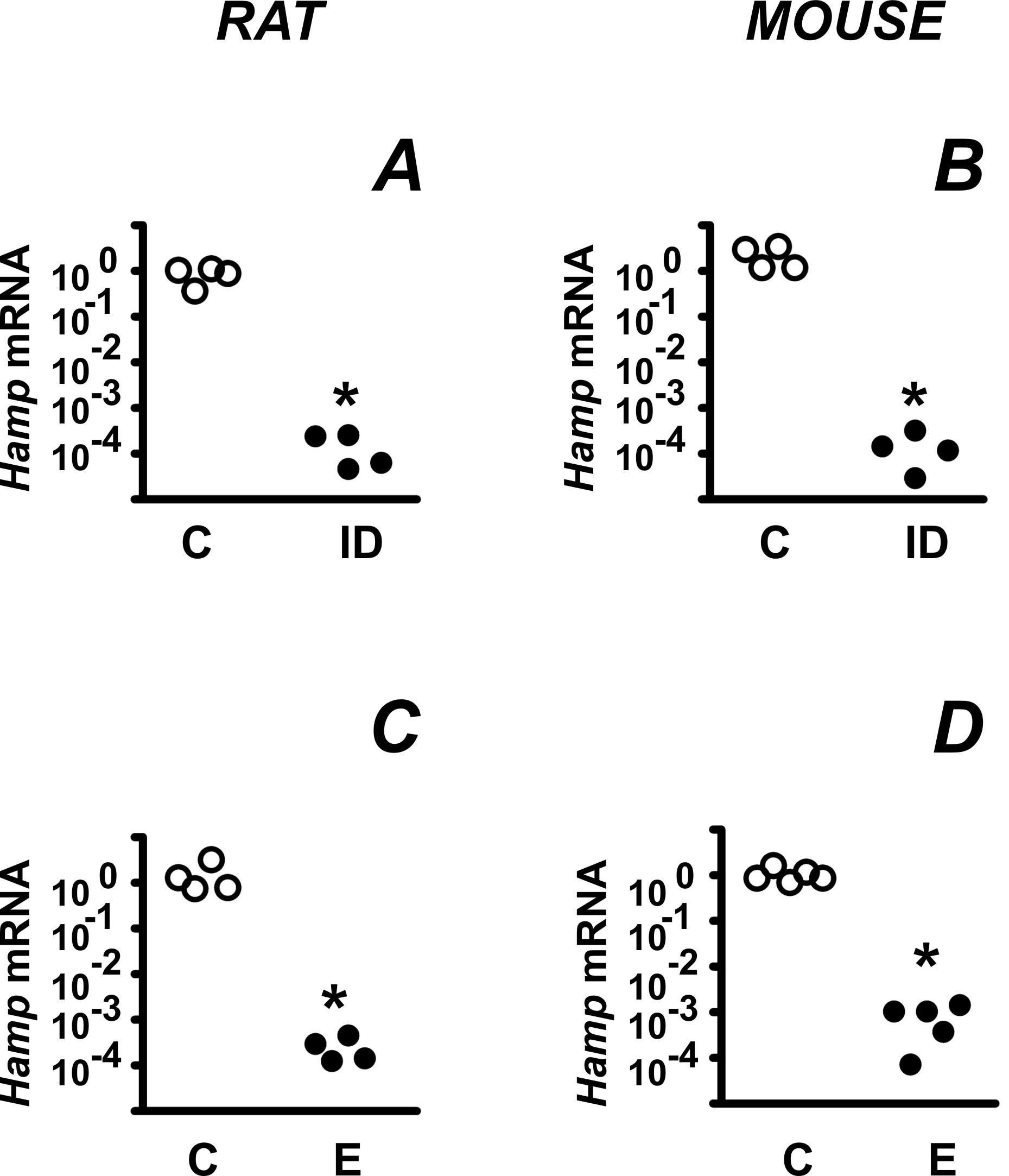
**
