# Supplementary material for: Effect of Erythropoietin, Iron Deficiency and Iron Overload on Liver Matriptase-2 (TMPRSS6) Protein Content in Mice and Rats
Source: PLoS One. 2016 Feb 4;11(2):e0148540. doi: 10.1371/journal.pone.0148540 (PMC4742081; doi:10.1371/journal.pone.0148540)

**S2 Fig. Lack of effect of a single dose of EPO on TMPRSS6 protein content in rats.**

(A) Immunoblot of TMPRSS6 and ATP1A (loading control) in the 3000 g fraction obtained from livers of female control rats (C) and rats administered a single i.p. dose of EPO at 500 I.U/rat 24 h before sacrifice. Liver iron content was 261 + 96 in the control group, and 207 + 14 in the EPO-treated group (n=4, p= 0.31).

(B) *Hamp* mRNA content in liver samples from the same animals. *Hamp* mRNA content is expressed relative to *Actb* mRNA, asterisk denotes statistical significance (*p*<0.05).


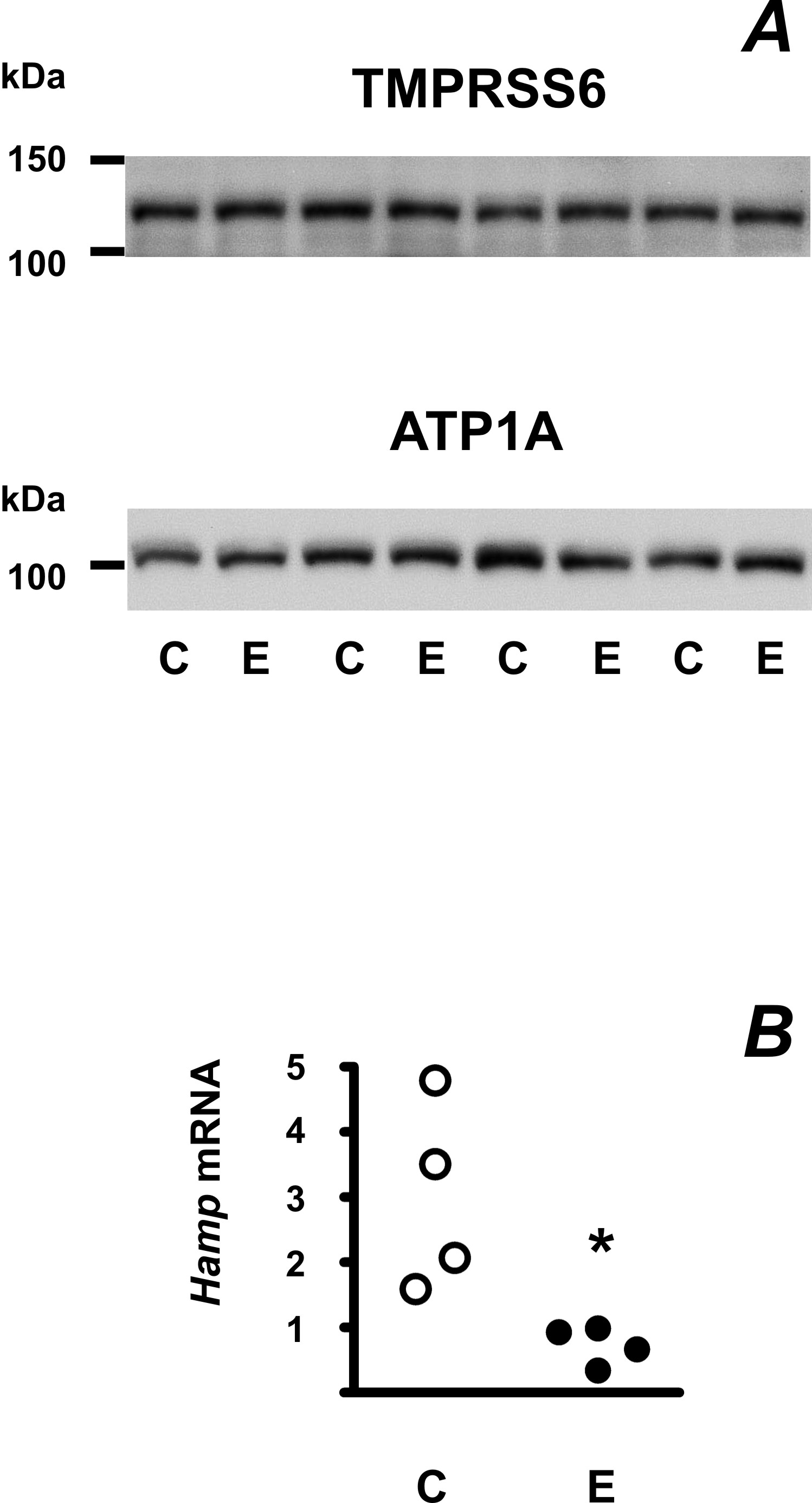

Supplement: S2 Fig — (DOC) [file pone.0148540.s002.doc]
