# Supplementary material for: Effect of Erythropoietin, Iron Deficiency and Iron Overload on Liver Matriptase-2 (TMPRSS6) Protein Content in Mice and Rats
Source: PLoS One. 2016 Feb 4;11(2):e0148540. doi: 10.1371/journal.pone.0148540 (PMC4742081; doi:10.1371/journal.pone.0148540)
Supplement: S3 Fig — (DOC) [file pone.0148540.s003.doc]

**S3 Fig. Lack of effect of low doses of iron-dextran on TMPRSS6 protein content in mice.**

Immunoblot of TMPRSS6 and ATP1A (loading control) in the 3000 g fraction obtained from livers of control male mice (C) and mice administered a single i.p. dose of iron dextran at 50, 100 and 150 mg/kg body weight one week before sacrifice.

**
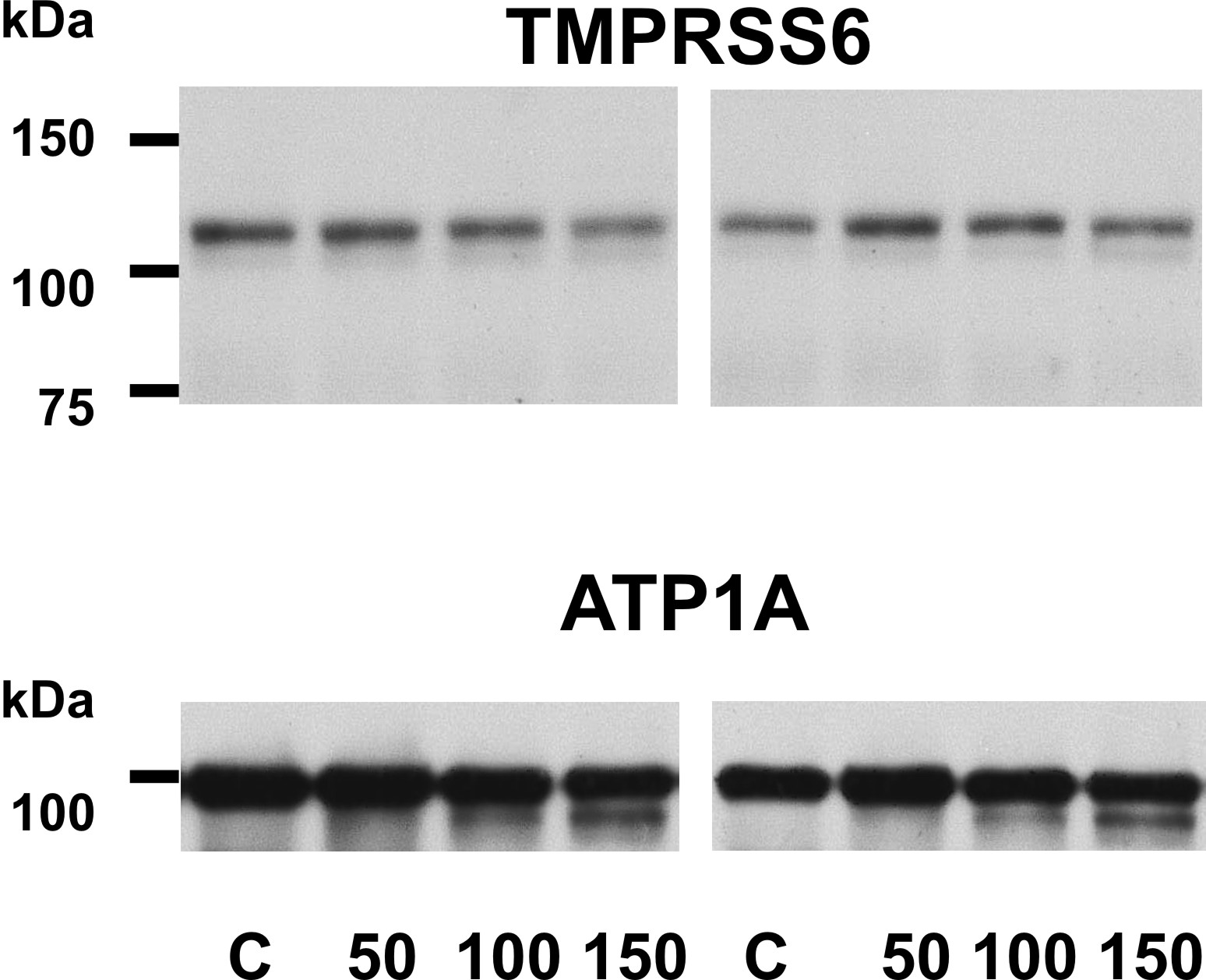
**
