# Supplementary material for: Effect of Erythropoietin, Iron Deficiency and Iron Overload on Liver Matriptase-2 (TMPRSS6) Protein Content in Mice and Rats
Source: PLoS One. 2016 Feb 4;11(2):e0148540. doi: 10.1371/journal.pone.0148540 (PMC4742081; doi:10.1371/journal.pone.0148540)
Supplement: S4 Fig — (DOC) [file pone.0148540.s004.doc]

**S4 Fig.** **Effect of iron on *Hamp*, *Bmp6* and *Tmprss6* RNA.**

(A-C): *Hamp* mRNA, *Bmp6* mRNA and *Tmprss6* mRNA content in livers of male C57BL/6 mice administered a single i.p. dose of iron dextran at 200, 350 and 750 mg/kg body weight one week before sacrifice. Target mRNA content is expressed relative to *Actb* mRNA. Data from three series of dose-response experiments.

(D-E): *Hamp* mRNA, *Bmp6* mRNA and *Tmprss6* mRNA content in livers of male C57BL/6 mice administered a single i.p. dose of iron dextran at 1000 mg/kg body weight 24 h before sacrifice. Target mRNA content is expressed relative to *Actb* mRNA. Asterisks denote statistical significance (*p*<0.05).

**
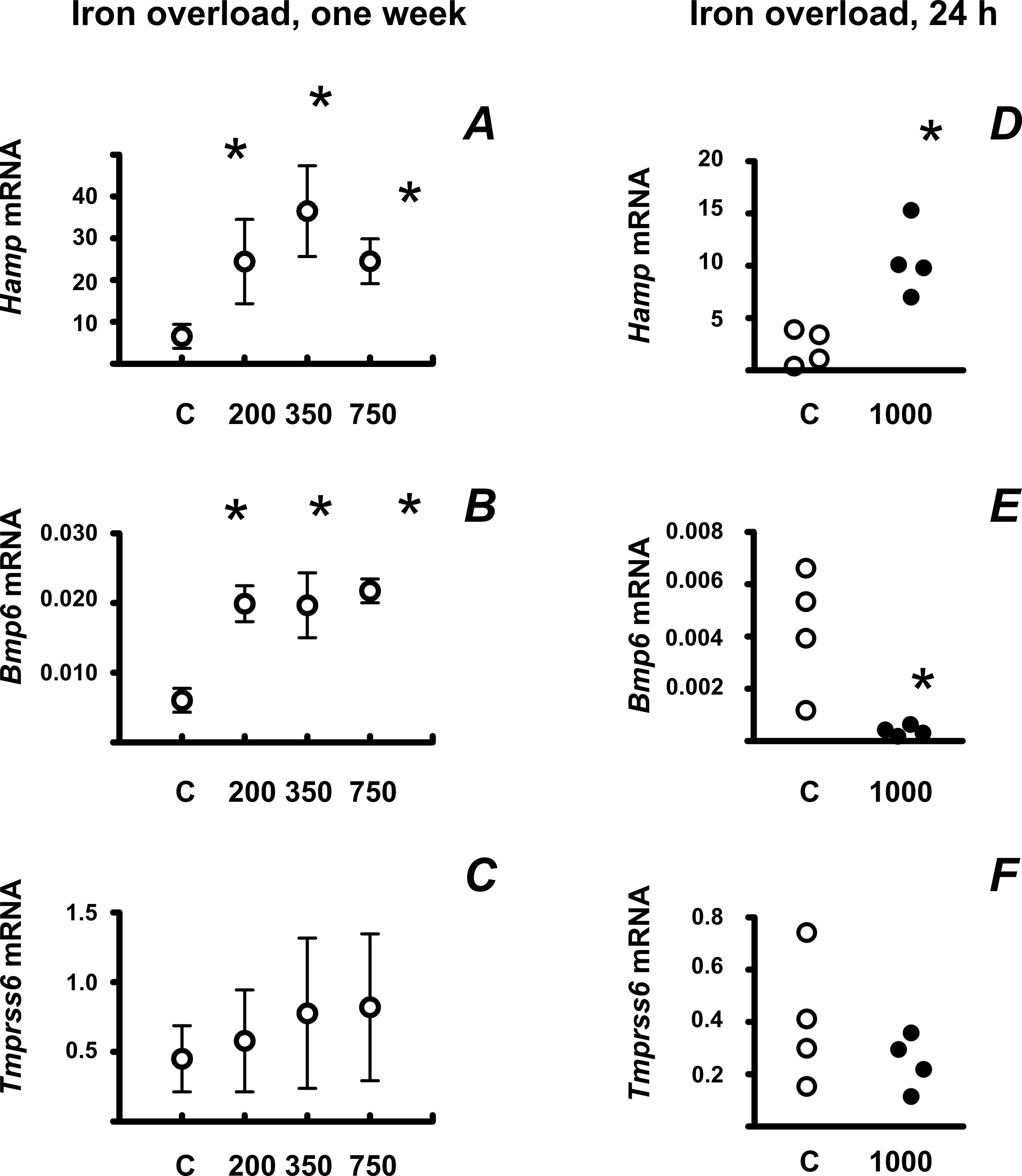
**
