# Supplementary material for: Effect of Erythropoietin, Iron Deficiency and Iron Overload on Liver Matriptase-2 (TMPRSS6) Protein Content in Mice and Rats
Source: PLoS One. 2016 Feb 4;11(2):e0148540. doi: 10.1371/journal.pone.0148540 (PMC4742081; doi:10.1371/journal.pone.0148540)

**S5 Fig**. **The 75 kDa band observed in mouse liver samples with high iron content is not TMPRSS6-specific.**

(A) Immunoblot of TMPRSS6 in the 3000 g fraction from liver of control mouse (C), mouse treated with 750 mg/kg iron (Fe), *Hjv*+/+ mouse (WT) and *Hjv*-/- mouse (KO). A strong 75 kDa band (arrowhead) is apparent in Fe sample and KO sample. Arrow denotes full length TMPRSS6 protein.

(B) Same samples treated with PNGase F. Arrows denote full length TMPRSS6 before and after PNGase F treatment (observed shift cca 29 kDa). Arrowheads denote the iron-induced 75 kDa band, which is not shifted following PNGase F treatment.

(C) Immunoblot of TMPRSS6 in the 3000 g fraction from liver of *Tmprss6*+/+ mice (WT) or *mask* mice (*m*) kept for four weeks on control (Con) or carbonyl iron (Fe) diet. Diet iron content was 2 %. Arrow denotes full length TMPRSS6 protein, arrowhead the non-specific 75 kDa band seen in Fe diet samples.


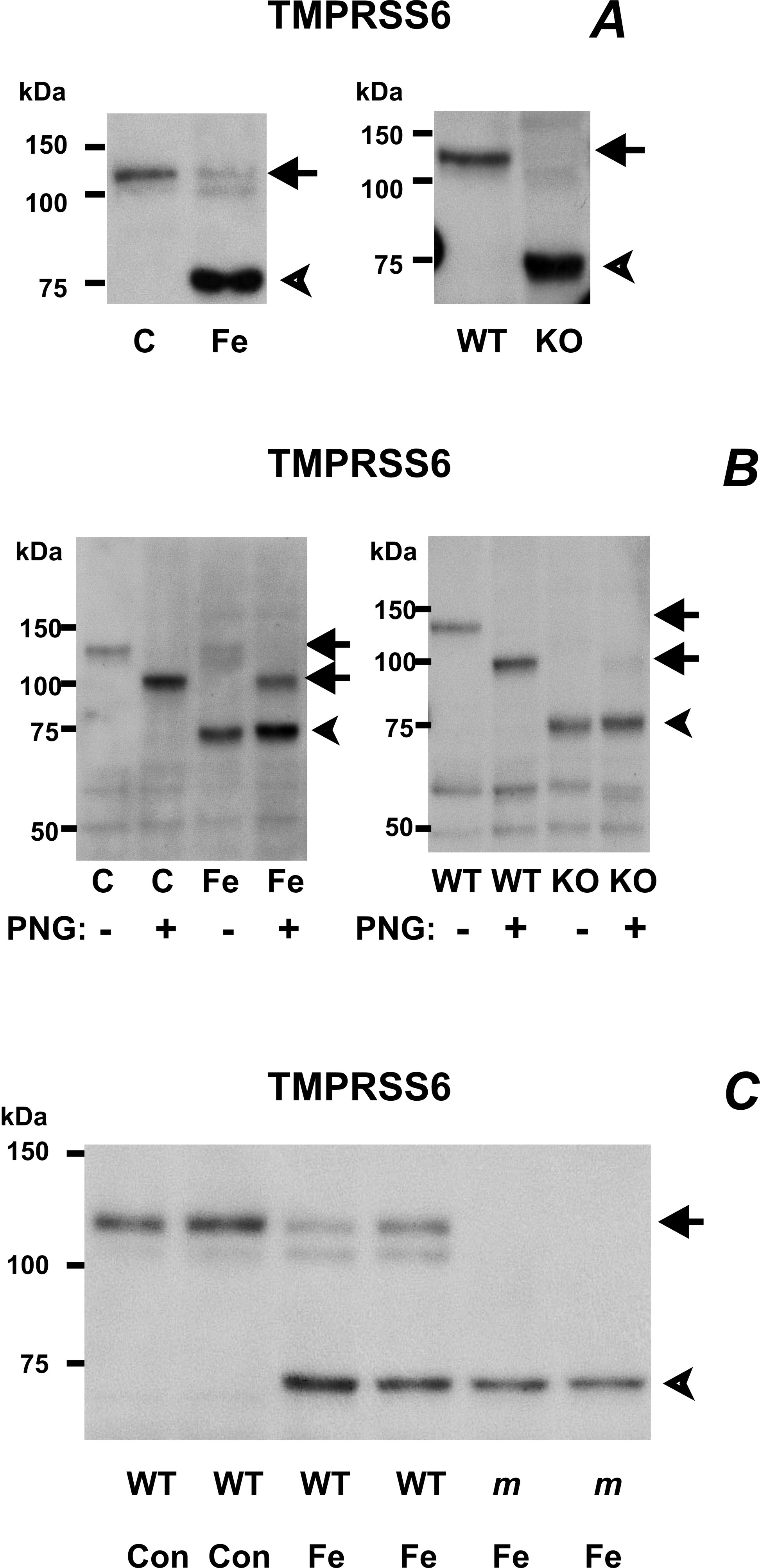

Supplement: S5 Fig — (DOC) [file pone.0148540.s005.doc]
