# Supplementary material for: Effect of Erythropoietin, Iron Deficiency and Iron Overload on Liver Matriptase-2 (TMPRSS6) Protein Content in Mice and Rats
Source: PLoS One. 2016 Feb 4;11(2):e0148540. doi: 10.1371/journal.pone.0148540 (PMC4742081; doi:10.1371/journal.pone.0148540)
Supplement: S6 Fig — (DOC) [file pone.0148540.s006.doc]

**S6 Fig. Lack of effect of EPO on TMPRSS6 protein content in *Hfe2*-/- mice.**

Immunoblot of Tmprss6 in the 3000 g fraction obtained from livers of *Hfe2-/-* mice and *Bmp6*-/- mice administered sterile water (C) or four daily doses of erythropoietin (E) at 50 IU/mouse. ATP1A is used as loading control.

**
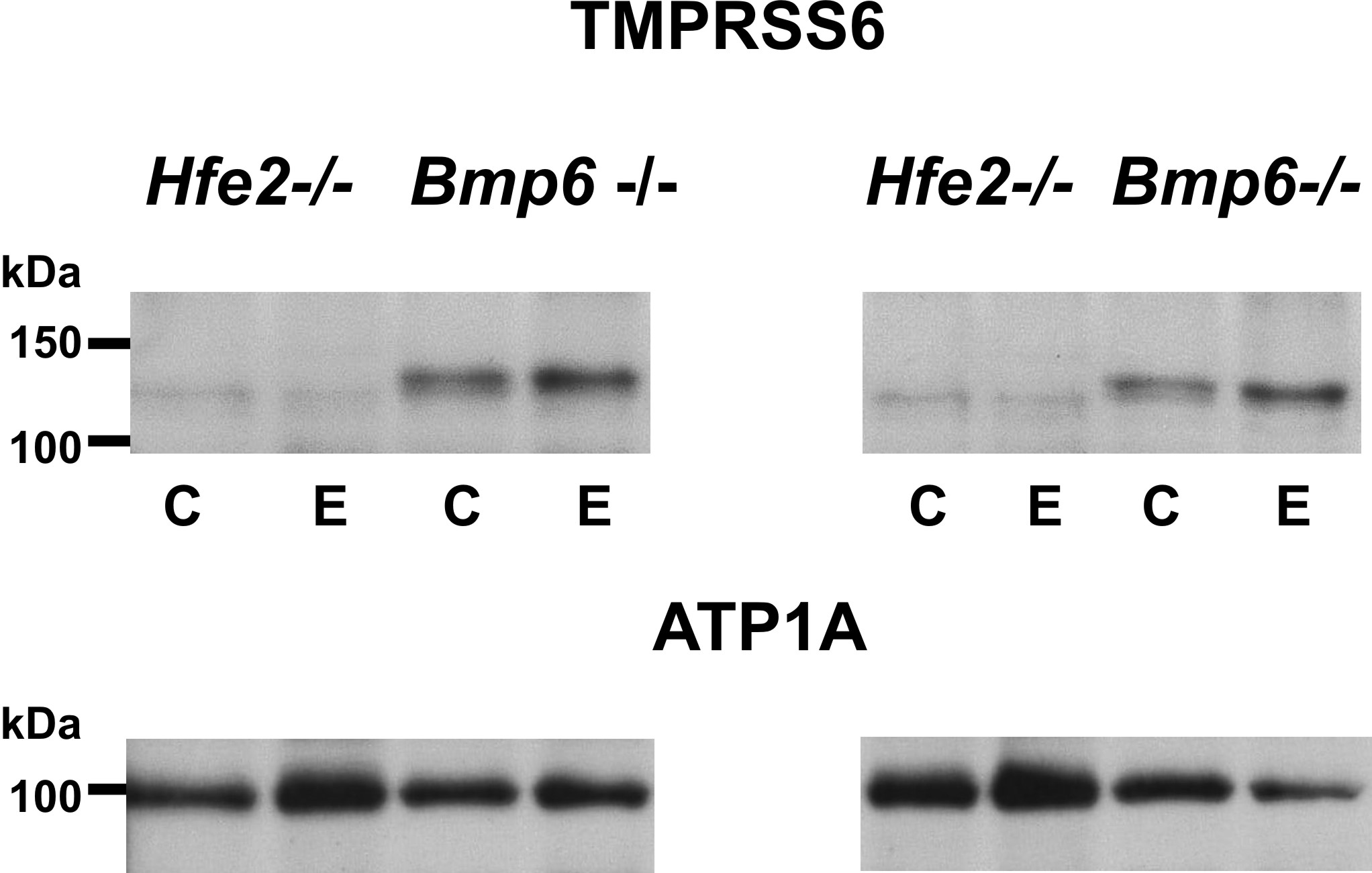
**
