# Supplementary material for: Effect of Erythropoietin, Iron Deficiency and Iron Overload on Liver Matriptase-2 (TMPRSS6) Protein Content in Mice and Rats
Source: PLoS One. 2016 Feb 4;11(2):e0148540. doi: 10.1371/journal.pone.0148540 (PMC4742081; doi:10.1371/journal.pone.0148540)
Supplement: S1 Table — (DOC) [file pone.0148540.s007.doc]

**S1 Table. List of primers used for PCR analysis**

Mouse *Actb*: GACATGGAGAAGATCTGGCA and GGTCTTTACGGATGTCAACG,

Mouse *Hamp*: CTGAGCAGCACCACCTATCTC and TGGCTCTAGGCTATGTTTTGC,

Mouse *Tmprss6*: GGTACCCTCTCTGGACTACGG and CAGAGCAGAGGAACTCACCA

Mouse *Bmp6*: GAACCTGGTGGAGTACGACAA and ATGCTCCTGCAAGACTTGGTA,

Rat *Actb*: TGTCACCAACTGGGACGATA and AACACAGCCTGGATGGCTAC,

Rat *Hamp*: GAAGGCAAGATGGCACTAAGCA and TCTCGTCTGTTGCCGGAGATAG,

Rat *Tmprss6*: GAAGAAGGGCTTGCATAGCTT and CCATAGTCCAGAGAGGGAACC.
