# Supplementary material for: Effect of Erythropoietin, Iron Deficiency and Iron Overload on Liver Matriptase-2 (TMPRSS6) Protein Content in Mice and Rats
Source: PLoS One. 2016 Feb 4;11(2):e0148540. doi: 10.1371/journal.pone.0148540 (PMC4742081; doi:10.1371/journal.pone.0148540)
Supplement: S2 Table — (DOC) [file pone.0148540.s008.doc]

**S2 Table. Liver iron content in iron-overloaded animals**

| Group and Treatment | Liver iron |
| --- | --- |
|  | μg/g wet wt. |
| Mouse, control | 38+2 |
| Mouse, 200 mg/kg i.p., 7 d | 1788+83 |
| Mouse, 350 mg/kg i.p., 7 d | 2697+125 |
| Mouse, 700 mg/kg i.p., 7 d | 3567+114 |
| Mouse, 1000 mg/kg i.p., 24 h | 6630+470 |
| *Hjv*+/+ mouse (female) | 163+17 |
| *Hjv*-/+ mouse (female) | 201+13 |
| *Hjv*-/- mouse (female) | 2527+70 |

Male C57BL/6 mice received one intraperitoneal injection of iron dextran 7 d or 24 h before sacrifice.. Data are expressed as mean + SD, n=3.
